# Modulation of Brain Hyperexcitability: Potential New Therapeutic Approaches in Alzheimer’s Disease

**DOI:** 10.3390/ijms21239318

**Published:** 2020-12-07

**Authors:** Sofia Toniolo, Arjune Sen, Masud Husain

**Affiliations:** 1 Cognitive Neurology Group, Nuffield Department of Clinical Neurosciences, John Radcliffe Hospital, University of Oxford, Oxford OX3 9DU, UK; masud.husain@ndcn.ox.ac.uk; 2 Wellcome Trust Centre for Integrative Neuroimaging, Department of Experimental Psychology, University of Oxford, Oxford OX2 6AE, UK; 3 Oxford Epilepsy Research Group, Nuffield Department Clinical Neurosciences, John Radcliffe Hospital, Oxford OX3 9DU, UK; arjune.sen@ndcn.ox.ac.uk

**Keywords:** Alzheimer’s disease, epilepsy, hyperexcitability, neurodegeneration

## Abstract

People with Alzheimer’s disease (AD) have significantly higher rates of subclinical and overt epileptiform activity. In animal models, oligomeric Aβ amyloid is able to induce neuronal hyperexcitability even in the early phases of the disease. Such aberrant activity subsequently leads to downstream accumulation of toxic proteins, and ultimately to further neurodegeneration and neuronal silencing mediated by concomitant tau accumulation. Several neurotransmitters participate in the initial hyperexcitable state, with increased synaptic glutamatergic tone and decreased GABAergic inhibition. These changes appear to activate excitotoxic pathways and, ultimately, cause reduced long-term potentiation, increased long-term depression, and increased GABAergic inhibitory remodelling at the network level. Brain hyperexcitability has therefore been identified as a potential target for therapeutic interventions aimed at enhancing cognition, and, possibly, disease modification in the longer term. Clinical trials are ongoing to evaluate the potential efficacy in targeting hyperexcitability in AD, with levetiracetam showing some encouraging effects. Newer compounds and techniques, such as gene editing via viral vectors or brain stimulation, also show promise. Diagnostic challenges include identifying best biomarkers for measuring sub-clinical epileptiform discharges. Determining the timing of any intervention is critical and future trials will need to carefully stratify participants with respect to the phase of disease pathology.

## 1. Introduction

Recent clinical and preclinical research has led to a growing realization of the strong association between brain hyperexcitability, manifest in its extreme form as epilepsy, and Alzheimer’s disease (AD) [1,2]. Epileptiform activity in AD might arise as a bystander effect, encountered as consequence of neurodegeneration as the disease progresses. On the other hand, it might be a constituent component of the AD phenotype [3,4]. It is now, for example, established that AD patients have higher rates of subclinical and overt epileptiform activity [2]. The prevalence of subclinical epileptiform activity is still largely unknown [5], with some evidence suggesting it could be present in up to 42.4% of AD cases [6]. Clinically overt seizures among AD patients have been reported to be from 6 to 17 times higher compared to age-matched controls [7,8,9], while the lifetime prevalence of seizures in AD populations ranges from 1.5 to 64%, partly owing to the pleomorphic clinical representations of epileptic discharges [10,11]. Most seizures are subtle and non-convulsive in AD; they could easily be missed, and confusional or amnestic episodes overlap with typical AD symptoms [12,13].

Preclinical data in both AD and epilepsy models show that covert epileptic discharges can have an adverse impact on cognition [14,15]. Murine models of epilepsy frequently report behavioural impairment in standard tests of spatial cognition such as the Morris water maze task [16,17], with a disruption of precise temporal organization of neuronal firing that is essential for normal cognitive processing [18]. Epileptiform discharges are also associated with impaired performance in cognitive tasks, usually involving memory and spatial processing in mouse models of AD [14,19]. Similarly, subclinical epileptiform activity in AD patients associates with an earlier and more rapid cognitive decline, in both memory and executive function [6,9].

While we focus on hyperexcitability, this is only one potential avenue to explore in the development of therapeutics for AD; other strategies are considered in detail elsewhere [20,21]. In this review, we consider the mechanisms—both at a systems network and molecular level—that might underlie hyperexcitability and its functional consequences in AD and discuss potential new therapeutic avenues that might target such hyperexcitability in clinical trials. We explore potential strategic pitfalls, which include controlling for genetic susceptibility and comorbidities such as vascular risk factors. We delineate optimal methods to detect sub-clinical epileptiform discharges. In addition, we review current evidence on ongoing clinical trials to evaluate the potential efficacy of established antiseizure medications (ASMs) as well as newer compounds and techniques targeting brain hyperexcitability in AD.

## 2. Network Dysfunction and Hyperexcitability

Excitability changes occur in several brain structures, and current evidence from animal and human models possibly points towards an early hyperactivity starting in the dentate gyrus [14,22], spreading to the hippocampus [14,23], and then to functionally and structurally connected brain regions, alongside AD disease progression [24,25]. Higher brain functions such as learning and memory depend on the interaction of a constellation of neurons, organized across multiple hierarchical levels, from local neuronal microcircuits to large long-range networks [26]. Covert epileptiform activity has been shown to disrupt micro and macro scale network function in patients with epilepsy and AD [6,27,28,29]. Indeed, both diseases are now conceptualized as brain network disorders [30,31], with AD recently labelled as a ‘disconnection syndrome’ [32]. Further, in addition to epileptic discharges acutely impacting upon cognition, the widespread inhibitory wave immediately following interictal epileptiform activity can reduce the power of gamma oscillations, associated with learning and memory function, in the hippocampus [33]. Dysfunction of single neurons observed in AD mouse models can impair long-range communication between distant brain regions, as indexed by the reduction of slow-wave oscillations and long-range coherence of neuronal activity across neocortical areas in amyloid precursor protein (APP) mouse models [34]. In patients with AD, slow-wave oscillations have recently gained attention for their key role in memory consolidation through cortico-hippocampal-thalamic coupling [35]. Their disruption during physiological sleep is thought to be linked to the development and worsening of memory deficits in AD [36,37], mediated by neuronal dysfunction associated with both Aβ amyloid and tau deposition [38,39].

What is the neurobiological substrate underlying this global disruption of neuronal firing? Epilepsy has been historically regarded as the prototype of an imbalance between physiological excitation and inhibition (E/I), where excitation prevails [40]. AD, increasingly viewed as a circuit-based disorder [1], is also associated with a disruption of the physiological E/I equilibrium [1,41]. Mouse models of AD support the concept that Aβ amyloid-induced change of the E/I balance initially causes hyperactivity in cortical and hippocampal neurons, a breakdown of slow-wave oscillations, as well as network hypersynchrony, even before the appearance of amyloid plaques [41]. Transgenic mice carrying either human APP [14,42] or presenilin-1 (PSEN1) mutations [43] display neuronal hyperexcitability, aberrant patterns of neuronal circuit activity and spontaneous seizure activity in cortical and hippocampal networks, with subsequent excitotoxicity and amplification of the synaptic release of Aβ [44]. Preclinical models of dual pathology, overexpressing Aβ and tau by crossing APP/PS1 and rTg4510 or rTg21221 mice, show that tau effects dominate and counteract Aβ-related hyperactivity, thus inducing neuronal silencing and hypoactive neuronal circuits later in the course of the disease [45].

**Figure 1:**
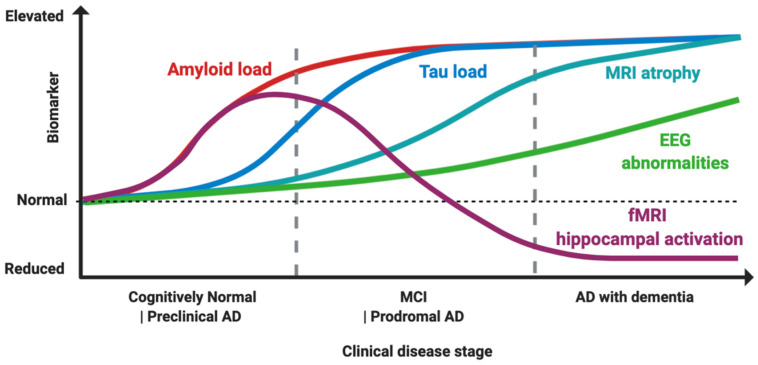
Proposed model of biomarker dynamics of hyperexcitability in humans. Amyloid load, measured by either cerebrospinal fluid (CSF) or Pittsburgh B compound amyloid ligand (PiB) positron emission tomography (PET), is the first to increase. Functional magnetic resonance imaging (fMRI) hippocampal activation is elevated in the preclinical and early prodromal Alzheimer’s disease (AD) phases, and subsequently decreases, with final hypoactivation in AD dementia stage. Tau load elevation, at CSF analysis or tau imaging, subsequently follows. Higher rates of MRI atrophy appear after fMRI hyperactivation and tau increase. Electroencephalogram (EEG) abnormalities increase longitudinally as disease progress, with suboptimal detection rates. The combined effect of Aβ amyloid and tau induces hyperexcitability in early and hypoexcitability in late disease stages, as depicted by fMRI hippocampal activation. Made in ©BioRender-biorender.com.

Evidence suggests that Aβ amyloid is associated with neuronal circuit *hyper*activity in earlier phases, mediated though both an increase in excitation and a decrease in GABAergic inhibition, and subsequently tau causes hypoactivation mostly through a decrease in excitation [45]. These effects together shift the normal E/I balance either towards *hyper*excitability or hypoexcitability, according to disease stage [1], (Figure 1). Importantly, suppression of the tau transgene in mice models of AD is not able to rescue E/I imbalance, suggesting that the damage caused by their pathological interaction might be irreversible under these conditions [45].

Hyperactivity also enhances pathological spread of toxic proteins, such as increasing Aβ diffusion and plaque deposition and promoting synaptic propagation of tau across different brain regions, with increased secretion, internalization and further seeding of additional tau [46,47], leading, ultimately, to neurodegeneration. As an example, in animal models such as the rTg4510 mice, hyperexcitability leads to increased tau pathology and cell loss, enhancing Ca2+ influx into neurons, and activating aberrant signalling cascades, including the activation of calcineurin-mediated pathways [47]. There is also evidence in humans with epilepsy that epileptiform activity itself can cause pathological accumulation of tau and increased rates of amyloidopathy [48,49]. Therefore, a disruption of the E/I equilibrium can have long-term effects in promoting neurodegeneration [1,50]. These findings have led to a growing appreciation that brain hyperexcitability is a key feature in AD, and hence, a potential target for therapeutic interventions aimed at enhancing cognition and, possibly, disease modification in the longer term by dynamic modulation of aberrant hyperexcitatory pathways [51]. Below, we first consider molecular pathways that might be potential targets for this strategic approach to treatment of AD.

## 3. Targeting Shared Molecular Pathways between Epilepsy and AD

**Figure 2:**
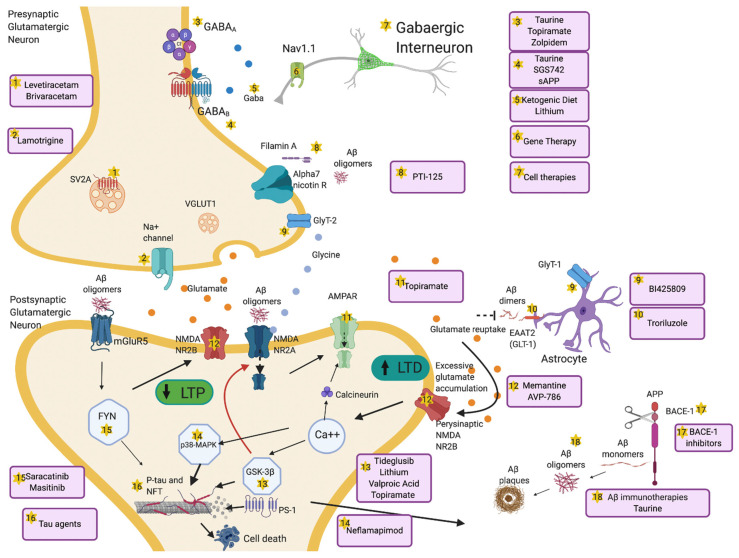
Overview of mechanisms and therapeutic targets of hyperexcitability in AD. Aβ dimers block glutamate reuptake by astrocytes through glutamate transporter-1 (GLT-1) receptors. This causes increased glutamate levels in the synaptic cleft, activation of perisynaptic N-methyl-D-aspartate (NMDA) 2B receptors, increased Ca++ influx, α-amino-3-hydroxy-5-methyl-4-isoxazolepropionic acid (AMPA) receptors internalization and activation of glycogen synthase kinase 3 beta (GSK-3β) and p38 mitogen-activated protein kinase (p38-MAPK) pathways. These pathologic cascades lead to abnormal tau phosphorylation and neurodegeneration. Long-term potentiation (LTP) is reduced and long-term depression (LTD) increases. Aβ oligomers interact pre- and postsynaptically with alpha-7 nicotinic receptors (alpha7-nAChRs), metabotropic glutamate receptors 5 (mGluR5s), and NMDA receptors. mGluR5 activates Fyn-mediated neurodegenerative changes. Increased excitation can also be driven by presynaptic changes in synaptic vesicle glycoprotein (SV2A) and Na+ channels. Decrease of GABAergic transmission or impaired glycine levels are also implicated in increasing hyperexcitability. Several therapeutic compounds are able to counteract specific molecular targets implicated in hyperexcitability. Made in ©BioRender-biorender.com.

To understand the potential links between epileptogenesis and neurodegeneration in AD it is important to gain insights into the fine balance between E/I changes at the synaptic level. In excitotoxic conditions, the frail physiological equilibrium between long-term potentiation (LTP) and long-term depression (LTD) appears to be disrupted, fostering neuronal hyperexcitability, which ultimately leads to a shift from synaptic equipoise [52]. In turn, this might be a key trigger for neurodegeneration [14,53]. Figure 2 provides an overview of potential mechanisms and therapies that might have an impact on molecular pathways implicated in epileptogenesis and AD.

### 3.1. Amyloid Aβ and Neurodegeneration through Epileptogenesis

As discussed above, Aβ amyloid might be one of the initial triggers to neuronal hyperexcitability in AD [54,55], particularly Aβ oligomers rather than amyloid plaques [56]. Such hyperactivity can be induced by direct application of exogenous Aβ amyloid into the brains of wild type mice and even through small elevations of endogenous Aβ amyloid [55,57], suggesting that a functional disturbance, rather than structural damage, is sufficient [41]. There is a strong correlation between brain Aβ amyloid load and the number of hyperexcitable cortical cells [58], with hyperactive neurons identified near amyloid plaques [59]. The preferential occurrence of hyperactive neurons in the vicinity of amyloid plaques might be related to the enrichment of oligomers in a plaques’ milieu [60]. Alternatively, plaques could develop preferentially near hyperactive neurons because of activity-dependent release of Aβ [61].

There is some evidence that Aβ amyloid can also act as a neuromodulator, altering synaptic facilitation and impacting on LTP [44]. Low levels of oligomeric Aβ reduce synaptic efficacy, while intermediate levels induce an optimal, physiological neurosynaptic facilitation, through activation of presynaptic alpha-7 nicotinic receptors (alpha7-nAChRs) and increasing probability of presynaptic vesicle release [44]. If Aβ oligomeric load increases further, however, excitatory neurotransmission is depressed through a range of mechanisms: internalization of synaptic N-methyl-D-aspartate receptors (NMADRs) and α-amino-3-hydroxy-5-methyl-4-isoxazolepropionic acid receptors (AMPARs) through calcineurin activation, activation of perisynaptic NMDARs, metabotropic glutamate receptors 5 (mGluR5s), and alpha7-nAChRs, which lead to impairment of LTP and facilitation of LTD, as well as spine loss in mouse models of AD such as Tg2576 and Swedish mutant APP (APPswe) [62,63]. Additional evidence from preclinical murine models of AD show that these changes cause activation of other LTD-related pathways such as p38 mitogen-activated protein kinase (p38-MAPK) and glycogen synthase kinase 3 beta (GSK-3β) processes which ultimately lead to neurodegeneration (Figure 2, labels 11,12,13,14,16). Importantly, Aβ-induced neuronal hyperexcitability in mouse models of AD and in human induced pluripotent stem cell (hiPSC)-derived AD neurons can still be rescued by β- and γ -secretase inhibition [55,58,64], suggesting that these aberrant maladaptive changes might still be reversible at this stage.

The E/I imbalance caused by Aβ oligomers appears to be caused not only by increased excitation, but also by reduced synaptic inhibition [45]. Evidence supporting reduced synaptic inhibition include reduced GABAergic terminals on cortical neurons proximal to Aβ plaques in preclinical and human models [65]; beneficial use of benzodiazepines to enhance inhibition and rescue neuronal hyperactivity in the APP23xPS45 mice model [22,59]; and restoration of cognitive function and Aβ toxicity in the APP/PS1 mouse model by GABA administration [66]. Further, overexpression of Nav1.1 (type I, alpha subunit) of sodium channels in parvalbumin-positive GABAergic neurons was shown to reduce hyperexcitability, rescue impaired gamma oscillations and improve cognitive deficits in the hAPP-J20 mice [67,68]. While there is overarching evidence of reduced number and neural activity of GABAergic neurons in multiple preclinical models of AD as well as in AD patients’ brains and cerebrospinal fluid (CSF), impaired GABAergic transmission in areas such as the CA1 could coexist with areas where GABAergic neurons are unaltered, or even increased [69]. Increased tonic GABA release by reactive astrocytes could exert diverse functional effects according to specific neuronal regions. In the dentate gyrus (DG), the majority of synapses near reactive astrocytes are glutamatergic, and therefore GABA release inhibits the activity of DG granule neurons, resulting in inhibition, reduction of spike probability and memory impairments [70]. Conversely, in cortical layers II–III, where GABAergic interneurons are widely distributed, GABA from reactive astrocytes could inhibit the activity of neighbouring interneurons, resulting in disinhibition of glutamatergic neurons and epileptiform discharges [70]. At a later stage, these changes appear to lead to increased compensatory inhibitory modulation at the network level [14,71].

Therefore, there is substantial evidence in preclinical models that Aβ production could be tightly linked to epileptiform activity, but what is the clinical evidence in humans of a correlation between seizures and increased Aβ amyloid load? Patients with late-onset epilepsy of unknown cause have higher prevalence of abnormal CSF Aβ_1–42_ and higher risk of progression to AD compared to healthy controls, suggesting a possible role for Aβ peptides in seizure pathogenesis in AD [72]. In a Finnish study in childhood onset epilepsy, epileptic patients had a substantially higher prevalence of abnormal Pittsburgh B compound amyloid ligand (PiB) binding on positron emission tomography (PET) imaging, roughly corresponding to the prevalence estimates in people a decade older in the control population [49].

One possible therapeutic implication is that by restoring physiological Aβ levels in patients with AD, it might be possible not only to prevent plaque formation, but also reduce spontaneous seizure activity. Several passive immunotherapies with anti Aβ amyloid agents (Figure 2, label 18) have been investigated in the past few years (Table 1). Although none have yet been granted a clinical license, on the basis of their effects on cognitive outcomes [73], there is good evidence that such therapies do reduce brain amyloid levels [74,75]. Unfortunately, Aβ immunotherapy clinical trials often have a history of seizures as an exclusion criterion and measures of brain hyperexcitability are not included as primary or secondary outcomes.

If Aβ acts as a neuromodulator, with both positive and negative effects on LTP depending upon Aβ concentration, it is also possible that a washout of amyloid could be detrimental, so analysis of when to halt Aβ clearance—the “tipping point”—might potentially cast light on optimal regulation of hyperexcitability. Recent studies of β-site amyloid precursor protein cleaving enzyme 1 (BACE-1) suggest that this might indeed be the case, and that this enzyme could be a key factor in epileptogenesis in AD [76], (Figure 2, label 17). Inhibition of BACE-1 proteolytic activity decreases Aβ generation and amyloid deposition, and thus has been an important focus in AD clinical trials, unfortunately with little therapeutic success [77], (Table 1). All BACE-1 inhibitors are able effectively to reduce Aβ levels in AD patients, with reduction rates ranging from 95 to 75% in the CSF [77]. However, they were not beneficial in phase 3 trials, with unfavourable side effects including worsening of cognition [78]. Understanding the failure of BACE-1 inhibitors provides a possible key to unravelling the complex balance occurring at the synaptic level in AD. Recent in vitro findings on the effect of different BACE-1 inhibitors on cortical neurons in rats, report that a low dose of Lanabecestat, which downregulated Aβ secretion to 30–50%, was able to avoid detrimental effects on synaptic transmission [79]. Conversely, high doses had a detrimental effect on synaptic plasticity [79].

### 3.2. Tau and Neurodegeneration through Epileptogenesis

Tau, whose interaction with Aβ is considered to be crucial in the pathological cascade of AD [80], also plays a role in neurodegeneration through epileptogenesis, and is mostly associated with neuronal hypoexcitability, in contrast to Aβ-mediated hyperexcitability [1,45]. Evidence from AD mouse models overexpressing tau, such as rTg4510, rTg21221 and P301S mice, suggest that neuronal hypoactivity is driven by soluble forms of tau rather than neurofibrillary tangles (NFT) [45,81], which can be rescued by switching off tau expression [82]. Several mechanisms have been proposed to modulate tau-induced hypoactivity in different mouse models, including disruption of neuronal firing patterns [83], reduced grid cell firing with spatial memory deficits [84], distal relocation of the axon initial segment [85], increased threshold for action potential firing and amplitudes of inward-rectifying potassium currents, and LTP reduction [86]. Hyperphosphorylated tau in dendritic spines is also able to interfere with glutamate receptor trafficking, and downregulate AMPAR and NMDAR subunits, thereby causing reduced neural transmission [84,87]. This appears to be achieved through mechanisms involving Fyn, a tyrosine-protein kinase involved in glutamate receptor trafficking and neuronal survival [88], and by Fyn-independent processes [89], (Figure 2, labels 11,12,13,14,15,16).

However, there is also preclinical evidence that tau *reduction* can lead to lower seizure susceptibility, and specifically through reduction of Aβ-induced hyperexcitability [19,88,90]. Increased seizure resistance and reduced cortical hyperexcitability though tau ablation was found in a tau knock-out mouse model [91] and was able to revert Aβ induced deficits in the APP mouse model [19]. Similarly, tau protein levels were directly correlated with seizure severity in mice, and reduction of tau through antisense oligonucleotides protected the animals against chemically induced seizures [92]. Either tau deficiency or expression of truncated tau are able to restore Aβ-dependent excitotoxicity and improve memory performance in a T maze task, therefore implying that the dendritic role of tau might explain this joint neurotoxicity [90]. Tau reduction is also able to prevent cognitive impairment, neuronal loss and death commonly observed in the APP23 mice model [90,93]. Notably, this has also been replicated in other animal models of dual pathology, such as the hAPP/Tau+/+ mouse model of AD [19,88], suggesting that prevention of Aβ excitotoxicity through targeting tau might protect against neurodegeneration. Moreover, hyperphosphorylated tau has been detected in brains of older patients with refractory epilepsy (without AD) specifically within the regions of epileptogenic cortex [94].

Bridging these apparently conflicting reports, of tau inducing hyper- and hypo-excitability, seems challenging, and a unifying explanation has yet to be found in the literature. Heterogeneous tau isoforms, recording techniques and preclinical models hinder comparability across studies, and evidence gathered from models of isolated tau pathology rather than dual pathology are even less easy to interpret. Different epileptiform events encompass decreased firing across different excitatory and inhibitory cell classes, and therefore, understanding of the complex network interactions is essential to interpret tau’s role in ictal and interictal discharges at the macroscale [95]. Macroscale epileptiform activity could still be associated with hypoactive neurons expressing pathological tau in localized small scale regions, and seizure suppression through tau reduction does not necessarily imply that tau directly causes neuronal hyperactivity [1].

Several anti-tau agents have now been investigated in AD [96], (Table 1). Active and passive immunotherapy with anti-tau antibodies in mice has proven beneficial in restoring cognition [97,98]. Active immunotherapies include AADvac1 [99], and ACI-35 [100]. According to the latest phase 2 trial on AADvac1, even if 80% of the patients developed anti-tau antibodies, and slowed the increase in blood neurofilament light chain (NFL) levels, there would be no improvement in cognition [101]. A phase 2A trial is currently ongoing to test ACI-35 in early AD patients. Passive immunotherapies include Gosuranemab (or BIIB092 or BMS-986168), Tilavonemab (or ABBV-8E12 or C2N 8E12), Semorinemab (or RO7105705), and Zagotenemab (or LY3303560). Most of these have received orphan drug status after failing to show significant effects, but phase 2 trials extensions are still ongoing (Table 1).

Salsalate, an acetylation inhibitor, is currently being tested in mild-moderate AD in a phase 1 trial (Table 1). LMTM (or Methylene Blue or TRx0237), an aggregator inhibitor, did not prove beneficial in large human trials in AD [102], even if subsequent analyses suggested a possible positive effect on rates of brain atrophy [103], and currently a phase 1b/2a is ongoing in mild cognitive impairment (MCI) and mild AD (Table 1). O- GlcNAcase (OGA) inhibitors, such as LY3372689, after yielding positive results in reducing tau pathology in mice [104], are currently being tested in healthy participants (Table 1). Microtubule stabilizers, such as Davunetide (or NAP) have not shown benefit in halting cognitive decline in MCI patients [105], (Table 1). Rolipram, a phosphodiesterase E4 (PDE4) inhibitor has been tested only preclinically, with encouraging results, as APP/PS1 mouse model of AD develops LTP deficits, but rolipram-treated APP/PS1 mice show comparable LTP induction to wild type mice [106,107], (Table 1).

Antisense oligonucleotides (ASOs) reducing tau expression have been able to protect against seizures in animal models [92], and were also able to revert memory impairments by reducing APP levels in the APP mouse model [108]. An excess of 4 compared to 3 domains in the microtubule binding region (MTBR) of tau is associated with tauopathies development [109], and ASOs have been used in mice to increase the number of 3 and lower the amount of 4 MTBRs [109,110]. However, until recently, their poor ability to cross the blood–brain barrier (BBB) has limited ASOs applications in humans [110]. A phase 1/2 study in patients with mild AD is currently ongoing to assess the safety and tolerability of intrathecally administered ASOs with BIIB080, which targets microtubule associated protein tau (MAPT) mRNA and decreases the amount of tau (Table 1).

GSK-3β, a principal enzyme responsible for pathological phosphorylation of tau [111], is upregulated in AD [112], (Figure 2, label 13), with its activity correlating with the amount of NFTs in AD brains [113]. Overexpression of GSK-3β causes hippocampal neurodegeneration [114] and learning impairment [115] and has recently also been shown to contribute to epileptogenesis [116]. Presenilin-1 (PS1) is a substrate of GSK-3β, (Figure 2, label 13). As both tau and GSK-3β bind to the same region of PS1, the ability of PS1 to bring tau and GSK-3β into close proximity suggests that PS1 may regulate the interaction of tau with GSK-3β. Mutations of PSEN1 in AD increase both the ability of PS1 to bind GSK-3β and enhance its tau-phosphorylating capability [117].

GSK-3β has also been implicated in synaptic plasticity, acting as conduit between LTP and LTD [118]: during LTD the transient activation of NMDA receptors leads to internalization of AMPA receptors from the surface of the neuron by GSK-3β [119], (Figure 2, label 11,13). After induction of LTP, GSK3β kinase becomes temporarily inactivated, leading to complete abolition of LTD, with LTP prevailing [120]. These considerations have led to proposals that therapeutic strategies involving GSK3β inhibition might beneficially boost LTP and depress LTD.

Inhibiting GSK-3β can restore cognitive function in tests of learning and memory, reduce Aβ production, plaque load, and tau phosphorylation in various mouse models of AD [121,122,123]. Crucially, GSK-3β inhibition seems to demonstrate anticonvulsant properties when the brain is in a hyperexcitable, pro-epileptic state [124].

Many different GSK-3β inhibitors have now been used in preclinical studies, including lithium and valproic acid [125], and members of the thiadiazolidinone family, among which the most promising compound has been NP-12 (Tideglusib). Tideglusib has led to reduced amyloid deposition, lower levels of tau phosphorylation, prevention of hippocampal damage, fewer memory deficits and show anticonvulsant activity in rodents’ models of AD and epilepsy [124,126], (Figure 2, label 13). After a promising phase 2a study in AD patients [127], a larger phase 2b study missed its primary endpoint [128], (Table 1).

Memantine and ifenprodil are selective NMDA NR2B antagonists which are also able to inactivate GSK-3β and reduce tau phosphorylation [129], (Figure 2, label 12), (Table 1). During normal synaptic activity NMDA channels are open for few milliseconds, and memantine is unable to act; instead during prolonged receptor activation, as in excitotoxic conditions, memantine is able to bind NMDA channels and block receptor activity, counteracting brain hyperexcitability [130]. Memantine can reverse Aβ-induced LTP deficits [131], lower Aβ pathological load, and increase synaptic density in the hippocampus of AD mouse models [132]. Interestingly, it also enhances protein phosphatase 2A (PP2A) activity, the principal tau dephosphorylating enzyme, that is severely dysregulated in AD [133]. Therefore, it is considered to be also an anti-tau agent, which could be important in preventing neurodegeneration through hyperexcitability reduction [134]. It is licensed for moderate-severe AD in several countries.

The src kinase Fyn may also bridge tau and amyloid pathology and is implicated in epileptogenesis. Fyn has been reported to become activated postsynaptically in response to the interaction between oligomeric Aβ and mGluR5 and thus to mediate Aβ toxicity [132], (Figure 2, label 15). Moreover, Fyn is able to phosphorylate tau, and often colocalizes with neurofibrillary tangles [135]. It also selectively increases NR2B trafficking and membrane stabilization, resulting in enhanced receptor transmission [136]. Saracatinib (or AZD0530) is able to block Fyn, restore synaptic depletion and spatial memory deficits in APP/PS1 mice [137], and increase hippocampal synaptic density [138], (Figure 2, label 15). A phase 2a clinical trial of Saracatinib in humans missed its primary endpoint and had a large dropout rate due to gastrointestinal side effects, even though there was a trend towards less shrinkage of the hippocampus and entorhinal cortex in the treated group [139], (Table 1).

A similar drug that has been shown to act on Fyn is Masitinib, a tyrosine kinase inhibitor, which also plays a role in neuroinflammation through targeting mast cells and macrophages and regulation of BBB permeability [140], (Figure 2, label 15). Encouraging data on a phase 2 study of Masitinib in mild-moderate AD showed improvements in tests of cognition, though with high rates of side effects, even if the majority of events were mild or moderate and transient [140]. Currently, a large phase 3 study in mild to moderate Alzheimer’s disease is ongoing, as add-on therapy to cholinesterase inhibitors and/or memantine, with the interim analysis results showing a positive trend of efficacy in one of the Masitinib doses tested [141], (Table 1).

## 4. Pro-Epileptogenic Neurotransmitters and Role of Antiseizure Medications

Several other mechanisms have been proposed to underlie the increased hyperexcitability in AD including increased glutamatergic tone, altered surface expression of postsynaptic AMPA and NMDA receptors, voltage-gated ion channels and impairment of GABAergic interneurons [4]. Preclinical evidence from mouse models shows that hyperactivation is initiated by the suppression of glutamate reuptake, through an Aβ-dependent blockage by Aβ dimers of glutamate transporter-1 (GLT-1) EAAT2, on astrocytes [56], (Figure 2, label 10). Astrocytes have a crucial role in controlling physiological glutamate diffusion and homeostasis of the synaptic cleft [56]. EAAT2 have been found to be pathologically reduced in AD patients in clinical and neuropathological studies, particularly in the hippocampus [146]. Aβ oligomers are also able to block neuronal glutamate uptake on neurons postsynaptically, increasing glutamate levels even further in the synaptic cleft [147]. This leads to excessive perisynaptic accumulation of glutamate and extrasynaptic NMDA NR2B activation (Figure 2, label 12), with subsequent increases in calcium levels and activation of p38-MAPK and GSK-3β pathways involved in hyperphosphorylation of tau and cell death [130], (Figure 2, label 13,14,16).

The decreased stimulation of excitatory synapses due to Aβ induces internalization of synaptic NMDA NR2A receptors, which in turn causes increased LTD. While synaptic NMDA NR2A receptors trigger LTP, both synaptic and extrasynaptic NMDARs are able to induce LTD [148] through increased calcium release and subsequent activation of calcineurin, which leads to internalization of AMPA receptors [149], (Figure 2, label 11). Aβ oligomers can also act presynaptically, forming complexes with alpha7-nAChRs, which induce increased levels of glutamate release [150], (Figure 2, label 8). Reduced levels of vesicular glutamate transporter 1 (VGLUT1) have been linked to reduced efficiency of glutamate metabolism, and have been described in animal models, as well as in patients with AD, correlating with memory impairment [151,152], (Figure 2).

Given all these possible therapeutic targets in pro-epileptogenic neurotransmitters, one might postulate whether ASMs might ameliorate cognitive deficits in AD. The answer is not straightforward because a delicate balance between reducing synaptic hyperexcitability and inducing synaptic depression might be necessary to improve cognition. Certain ASMs also have known cognitive side effects, and therefore should be potentially avoided in AD: benzodiazepines, carbamazepine, eslicarbazepine, oxcarbazepine, phenobarbitone, phenytoin, primidone, tiagabine, topiramate, valproate, vigabatrin, and zonisamide [153].

Older ASMs, such as phenobarbitone, oxcarbazepine, carbamazepine, and valproate, have high rates of cognitive side effects, including sedation, somnolence and confusion [154]. Pre-clinical data in the APP/PSEN1 mouse model of AD show that sodium channel blockers, such as carbamazepine, phenytoin, and valproic acid, are able to reduce the frequency of spontaneous electroencephalogram (EEG) epileptiform discharges, with valproic acid being the most effective [155]. Valproic acid has also been shown to inhibit GSK-3β activity [156], lower Aβ production, reduce neuritic plaque formation and improve memory and behavioural deficits in AD mouse model [157], (Figure 2, label 13). Despite the promising pre-clinical data, valproic acid, has shown no clinical benefit in controlling behavioural symptoms in the treatment of agitation or psychosis in AD, and in fact, has been associated with higher rates of adverse effects, such as somnolence, tremor, faster decline in cognitive test scores, and greater brain volume loss on magnetic resonance imaging (MRI) [158,159]. Eslicarbazepine seems to have less cognitive side effects compared to carbamazepine and oxcarbazepine [160,161], but is still not frequently used in clinical practice in AD patients [153]. Primidone has shown a potential to exacerbate dementia, and therefore should be avoided [162].

Benzodiazepines, even if able to revert Aβ toxicity in preclinical models [34,59], are relatively contraindicated in older people, and particularly in patients with dementia owing to their potential to exacerbate confusion [163]. Other GABAergic drugs include vigabatrin, which can associate with irreversible visual field loss, and tiagabine, which is rarely used as drug of choice in AD patients [153]. Pregabalin and gabapentin have shown a potential in treating agitation and aggression in AD patients, even if evidence from large trials is still lacking [164], and cognitive side effects even in healthy people are reported with pregabalin [165].

Topiramate inhibits GSK-3β activation and histone deacetylase activity, inhibits Na+ and Ca2+ channels, enhances GABA_A_ receptor function, and blocks AMPA and kainate receptors (Figure 2, label 3,11,13). Despite promising preclinical data in the APPswe/PS1dE9 transgenic mice [166], topiramate has been consistently associated with poor cognitive performance in humans, and specifically shown to be worse than other drugs in its class, such as zonisamide [167].

The ASM lamotrigine acts by binding voltage-gated sodium channels, stabilizes presynaptic neuronal membranes and inhibits glutamate release [168], (Figure 2, label 2). Promising data in the APP/PSEN1 mice show that chronic treatment with lamotrigine is associated with reduced number and size of amyloid plaques in the cortex and hippocampus, and restoring of synaptic plasticity, learning, and memory deficits [169]. Lamotrigine has a favourable profile in the treatment of epilepsy in AD, with high efficacy on seizure control and tolerability, as well as positive mood stabilizing effect [170]. It has also been shown to improve performance on recognition and naming tasks in AD [171], (Table 2). Nevertheless, the risk of myoclonus with lamotrigine may need to be considered, as it can theoretically exacerbate the myoclonus observed in some AD patients, especially those with PSEN1 mutations, even if the risk in generalized epilepsy is relatively low [172].

Data on the use of lacosamide in AD is lacking, but generally has been associated with few cognitive side effects in a number of small studies [173,174], and is well tolerated even in older people with epilepsy [175]. Lacosamide also has a potential benefit in controlling behavioural and mood symptoms in other types of dementia [176,177].

Similarly, no data on perampanel cognitive side effects in AD are available, but few studies do no report worsening of cognition in patients with epilepsy in standard tests of cognition, but also found no improvement [178,179].

There is now considerable interest in levetiracetam and similar compounds to help treat hyperexcitability in AD. Levetiracetam, is an ASM that acts presynaptically, binding to the synaptic vesicle glycoprotein 2A (SV2A) [180], (Figure 2, label 1). It has been shown to reduce epileptiform discharges, lower hippocampal hyperactivity in animal models [181,182], suppress neuronal network dysfunction, decrease Aβ plaque burden and reverse cognitive deficits in AD models [166,183]. Moreover, in patients with amnestic MCI, low doses of levetiracetam have been shown to reduce hippocampal functional MRI (fMRI) hyperactivity in the dentate gyrus/CA3 region [184,185] and improve cognition in a pattern separation and completion task in amnestic MCI patients [22].

Good efficacy in reducing seizures, favourable tolerability and improved cognition with levetiracetam has been reported in AD [170,186]. Evidence from a randomized 3-arm case-control study on AD patients with epilepsy showed that levetiracetam led to better cognitive performance compared to phenobarbital and lamotrigine, with comparable levels of efficacy in reducing seizures in 58–71% of the patients and achieving seizure freedom in 24–28% of the cases [187]. To date, seven different phase 1 or 2 clinical trials have been registered to systematically investigate the use of levetiracetam in AD patients (Table 2).

Brivaracetam, like levetiracetam, binds SV2A, but with 15–30 fold higher affinity [184], (Figure 2, label 1). Unlike levetiracetam, it exerts no direct effect on AMPA, GABA, glycine, or kainic acid-gated currents, and has only a minor inhibitory action on NMDA receptors’ activity at supratherapeutic concentrations [188]. Brivaracetam was also shown to reduce spike-wave discharges and reverse memory impairments in the APP/PS1 mice model, though these were not associated with changes in Aβ metabolism or deposition [182], (Table 2). Notably, in that study both brivaracetam and another ASM, ethosuximide, reduced spike-wave discharges, but only brivaracetam reversed memory impairment. Importantly, ethosuximide has a completely different mode of action to all other ASMs, as it blocks T-calcium channels, and would likely not be prescribed in AD as it is specifically a drug to treat absence seizures, often in children [189,190].

Three other important compounds have been studied in AD for their ability to modulate NMDA receptors. The first one is lithium, which has multiple mechanisms of action, such as downregulating NMDA receptor activity and increasing GABAergic transmission, and has been shown to inhibit GSK-3β, reduce tau phosphorylation, lower Aβ production and restore memory deficits in AD transgenic mouse models [191], (Figure 2, label 5, 13). A recent metanalysis in patients with MCI and AD showed that lithium significantly inhibited the progression of cognitive decline with moderate effect size and comparable side effects compared to placebo, though with no change in CSF biomarkers (Aβ_1–42_, total tau (t-tau) and hyperphosphorylated tau (p-tau)) [192]. A clinical trial is currently ongoing to assess prevention of cognitive decline in patients with MCI (Table 2). Nevertheless, given its narrow therapeutic range and multiple drugs interactions [193], clinicians are likely to be reluctant to prescribe lithium to patients lacking capacity.

The second one is BI425809, a glycine transporter inhibitor [194]. Glycine is an NMDA receptor co-agonist, and glycine transporters GlyT-1 and GlyT-2, located respectively in astrocyte and neuron presynaptic membranes, take up glycine into the nerve terminal and glial cells, thus modulating glycine levels in the synaptic cleft [195], (Figure 2, label 9). BI425809 blocks these receptors, increases glycine levels and boosts NMDA receptor function. Nevertheless, a large phase 2 clinical trial missed its primary and secondary endpoints, showing no improvement in cognition [196], (Table 2). The third drug is AVP-786, a small molecule containing dextromethorphan, usually found in cough syrups and quinidine preparations. Dextromethorphan is a weak antagonist of NMDA receptors, and is currently being evaluated in two phase 3 trials in moderate AD with clinically significant agitation, the first one having reported preliminary negative results, while the second met its primary endpoints [197], (Figure 2, label 12), (Table 2).

Another attempt to restore glutamate homeostasis comes from the use of a prodrug of riluzole, Troriluzole (or BHV-4157). It acts by increasing the expression and function of the glial GLT-1, which lowers the levels of glutamate in the synaptic cleft and reduces glutamate-induced excitotoxity [198], (Figure 2, label 10). A phase 2/3 trial in AD patients is ongoing and has recently passed the interim futility analysis (Table 2).

A completely distinct therapeutic strategy is modulating GABAergic transmission. Aβ suppresses synaptic inhibition via downregulation of GABA_A_ receptors [199]. GABA_A_ receptor agonists have been successful in rescuing memory impairment, LTP deficits, and reducing hyperexcitability in the hippocampus in various preclinical models of AD [69]. Zolpidem is a GABA_A_ receptor agonist, and while there in an ongoing phase 3 clinical trial to assess its efficacy in improving sleep disorders in AD patients, it has also been linked to increased risk of developing AD [200], (Figure 2, label 3), (Table 2). Different compounds belonging to the same family of taurine, such as homotaurine (or vivimind or tramiprosate or alzhemed), and ALZ-801 act as GABA_A_ receptor agonists and GABA_B_ antagonists [201]. They also inhibit the interaction between Aβ and endogenous glycosaminoglycans, thus preventing fibril formation [202], (Figure 2, label 3, 4, 18). After a phase 3 failure in 2007, tramiprosate was repurposed and branded as a nutritional supplement [203], (Table 2). In 2017, after almost 10 years, a sub-analysis showed a potential benefit in slowing cognitive decline in ApoE4 homozygotes, especially in mild AD [204], with lower rates on hippocampal atrophy in ApoE4/4 carriers treated with tramiprosate [205].

Pathologically reactive astrocytes induce excessive tonic GABA secretion, which binds to neuronal GABA_B_ receptors at extrasynaptic sites, inhibiting synaptic release in APP/PS1 AD mice [70]. GABA_B_ receptor antagonists have been shown to ameliorate Aβ-induced learning, memory, and cognitive impairments in mice, rats and in Rhesus monkeys [69]. SGS742 (or CGP36742), a GABA_B_ antagonist, has shown beneficial effects on cognition in a phase 2 clinical trial in MCI patients [206], and another in patients with mild-moderate AD was subsequently started (Figure 2, label 4), (Table 2). More recently, sAPP, the soluble amyloid precursor protein, has shown to be able to modulate GABA_B_R1α activity, reducing synaptic activity and enhancing LTP, possibly paving the way to new pharmacological interventions to counteract neuronal hyperexcitability [207], (Figure 2, label 4), (Table 2).

Another way of increasing GABAergic transmission comes from ketogenic diet. A recent metanalysis examined the current evidence on KD in AD [208]. The core characteristic of the KD is the association of a high amount of fat with low carbohydrate intake, which leads to the production of ketone bodies to fuel the brain in the absence of glucose. It is a validated treatment for pharmacoresistant epilepsy [209]. Several neuroprotective effects have been observed with KD including protective effects against cerebral Aβ toxicity in the hippocampus in a rat model [210,211], (Figure 2, label 5). Similarly, in human studies, there was a significant improvement in cognitive outcomes (global cognition, memory and executive functions) with either supplementation of ketone bodies or KD in MCI and AD patients [212,213]. Unexpectedly, the antiepileptogenic mechanism of action of KD is rarely mentioned in these studies, with most of the rationale focusing on insulin resistance in AD [214]. However, the diet is also associated with significant weight loss, which is a key limitation especially in an ageing population [211]. Several trials are now ongoing in MCI or AD to assess the feasibility of the KD or ketone body supplements in patients with MCI or AD (Table 2).

Two new ground-breaking strategies have focused on potentiating GABAergic neuronal function. The first is represented by the use of hiPSCs, which can be induced to differentiate into mature cell subtypes, such as GABAergic neurons [215]. Stem cells have been used to replace dysfunctional GABAergic interneurons in various mouse models of AD, as they functionally integrate into existing pathological circuitries, replenish the lacking GABAergic tone, and lead to behavioural improvement in learning and memory [216,217]. There is evidence that transplanted cells are able to develop into mature interneurons, functionally integrate into the hippocampal circuitry and rescue learning and memory in ApoE4 knock-in mouse models, despite the toxic environment created by ApoE4 alone or in combination with Aβ [218]. Several phase 2 studies with stem cells in AD are currently ongoing [219], (Figure 2, label 7), (Table 2). Another promising approach for an engineered recovery of GABAergic transmission might be gene therapy, especially targeting Nav1.1, the voltage-gated sodium channel subunit predominantly expressed in interneurons (Figure 2, label 6). There is preclinical evidence that hypofunction of Nav1.1 could be restored by γ-secretase inhibitors (Semagacestat or LY450139) [220] and that Nav1.1-overexpressing interneuron transplant is able to enhance gamma oscillatory activity, reduce network hypersynchrony, and improve cognitive functions in the human APP transgenic mice [67]. Unfortunately, three phase 3 trials in AD patients failed because of an increased risk of skin cancer and infections and worsening of cognition [221], (Table 2).

## 5. Contribution of Vascular Mechanisms and Neuroinflammation to Epileptogenesis

### 5.1. Cerebrovascular Risk Factors

Vascular damage can play an important role in epileptogenesis. A higher load of vascular risk factors or changes on neuroimaging increases the likelihood of seizures in AD [222,223]. Conversely, patients with epilepsy have higher rates of cerebrovascular disease [224], and hypertension is an independent risk factor for epilepsy [225]. Therefore, the interrelationships between the two are complex and bidirectional [226]. Disentangling the three-way interaction between vascular damage, epilepsy and AD is even more daunting, given that AD and small vessel cerebrovascular disease (SVD) can often coexist in mixed dementia patients [222]. They frequently share common risk factors, such as hypertension, diabetes, obesity, smoking, and reduced physical activity [2].

One question that remains to be answered is whether aggressive management of such vascular risk factors in patients with AD might prevent epileptiform activity and, in the longer term, impact positively upon cognitive decline. In a large in vitro study in an AD transgenic mouse model, seven different antihypertensive medications seemed to reduce Aβ accumulation, whereas in a subsequent study, only Valsartan lowered the oligomeric form of Aβ [227,228], (Table 3). Several different classes of anti-diabetes drugs have also been evaluated for the treatment or prevention of AD. The most promising results come from Dapagliflozin, which acts by inhibiting the sodium-glucose cotransporter-2 (SGLT2). In diabetic rats and mice, dapagliflozin improved cognition, as well as brain mitochondrial function, insulin signalling, neurogenesis, synaptic density, and hippocampal synaptic plasticity [229]. From epidemiological data, it seems that use of SGLT2 inhibitors and other anti-diabetes drugs reduces the risk of dementia in diabetics [230]. Clinical trials are currently ongoing to evaluate the effect of dapagliflozin on cognitive function in patients with Type 2 diabetes mellitus (DM), and brain metabolic markers in AD patients with and without Type 2 DM (Table 3).

Given the poor crossing of the BBB by most compounds involved in hyperexcitability, alternative routes, such as the intranasal route have been proposed for drug delivery in AD [231]. This strategy has shown some promise in counteracting excitotoxicity as a result of overactivation of NMDARs in preclinical models of stroke [232,233]. Intranasal insulin has received significant attention, as it avoids the unwanted effect of increasing systemic insulin levels, leading to potential hypoglycaemia or insulin resistance [234]. AD has sometimes been referred to as type 3 diabetes, and administration of intranasal insulin could result in increase of brain glucose, reduced insulin resistance, neuroinflammation, and oxidative stress [235]. There is evidence of a possible beneficial impact on cognition and an increase of Aβ 40 levels in plasma in amnestic MCI and mild AD [234,236]. Moreover, reduced MRI atrophy and tau-P181/Aβ42 ratio have been reported with intranasal insulin administration [237]. However, a multicentric phase 2/3 clinical trial of intranasal insulin on 240 patients with MCI and AD has recently reported negative results [238]. Other clinical trials are currently ongoing to evaluate the long-term effect of nasal insulin in MCI or AD patients, and their bioavailability in the central nervous system (CNS) (Table 3).

The role of altered lipid metabolism in AD has gained attention since the discovery of ApoE4 genotype as major risk factor for late onset AD [239]. AD brains contain truncated, neurotoxic forms of ApoE4, in which the lipid binding domain mediates neurotoxicity [239]. Besides the role of ApoE4 in mediating neuronal hyperexcitability [240], lysophosphatidic acid (LPA), a synaptic phospholipid, has also been implicated in regulating brain E/I balance [241]. LPA is the major bioactive component of oxidized low-density lipoproteins (oxLDLs), which are important in atherosclerotic plaque formation, but also enhance Aβ production in cell cultures with wild type presenilin 1 (PS1wt) and APPswe mutations through upregulation of BACE-1 [242]. LPA also increases GSK-3β activity and subsequent tau phosphorylation [243] as well as promoting neurite retraction through activation of p38-MAPK [244]. Mice lacking LPA1 receptors have hippocampal deficits associated with behavioural impairments, such as impaired spatial memory retention and altered exploration [245]. Moreover, LPA-synthesizing enzyme autotaxin (ATX) is expressed in the astrocytic compartment of excitatory synapses and modulates glutamatergic transmission [246].

Dysfunctional expression and activity of ATX with associated changes in LPA signalling have recently been implicated in the pathogenesis of AD [247]. Higher levels of ATX have, for example, been found in MCI and AD patients and correlated with hypometabolism at fluorodeoxyglucose (FDG)-PET in medial temporal lobe, lower scores on tests of executive function and memory, reduced cortical thickness in the prefrontal cortex and CSF biomarkers of AD [246]. Pharmacological inhibition of ATX was able to reverse cortical excitability in a mouse model of schizophrenia [241], but no data on AD preclinical model are available. Therefore, further data are needed to explore the potential of LPA-ATX modulation in AD.

### 5.2. Neuroinflammation

Neuroinflammation and especially the role of interleukin 1 beta (IL-1β) in promoting epileptogenesis and neurodegeneration has gained increasing attention in both AD and epilepsy [248,249]. IL-1β levels are elevated in AD brains and correlate with β-amyloid plaque progression [250,251]. There is evidence of a vicious circle whereby seizures can cause neuroinflammation, with an overexpression of IL-1β, tumour necrosis factor alpha (TNFα), and interleukin 6 (IL-6), which in turn increase seizure severity, and cause downstream cognitive effects such as BBB disruption, inhibition of hippocampal LTP [252,253], and neuronal death [254]. Moreover, inhibiting IL-1β has been shown to have beneficial effects on cognition in rats [255]. The reduced BBB penetration of Anakinra, an IL-1 receptor antagonist, and canakinumab, a IL-1β neutralizing antibody, have limited their application in AD [256], and might also be why most of the clinical data on the use on nonsteroidal anti-inflammatory drugs (NSAIDs) in AD yielded negative results [257]. Nevertheless, a more tailored approach could possibly be beneficial.

Neflamapimod (or VX-745), a p38-MAPK inhibitor involved in formation of tangles and in microglial release of pro-inflammatory cytokines, such as TNFα and IL-1β, has recently shown positive effects on cognition in two open label phase 2 trials in mild AD [258], while a bigger randomized and blinded phase 2 trial was negative, and a fourth phase 2 trial is currently ongoing (Table 3), (Figure 2, label 14). Another molecule implicated in neuroinflammation and AD pathogenesis is Filamin A (Figure 2, label 8). This protein stabilizes the pathological interaction between Aβ amyloid and alpha7-nAChRs. A filamin inhibitor, PTI-125, has been recently studied in AD, and has proven to reduce tau phosphorylation, amyloid deposition, neuroinflammation and improve cognition in a mouse model of AD [259]. A phase 2 clinical trial in mild-moderate AD showed decreased CSF levels of t-tau, p-tau, IL-6, IL-1β, and TNFα [260], and another phase 2 trial is currently ongoing (Table 3). Notably, filamin blockage is thought to be beneficial in various models of epilepsy [261].

## 6. Who, When, and How to Treat Brain Hyperexcitability: Diagnostic and Therapeutic Challenges

Preclinical and human studies show that seizure susceptibility is higher if a genetic risk factor for early or late onset AD is present [42,44,262,263]. Young patients who carry APP, PSEN1, or PSEN2 mutations show an increased prevalence of seizures compared to sporadic AD patients [11], which could be as high as 87 fold [264]. ApoE4+ mice show increased hyperexcitability, especially in the entorhinal cortex, even independently of Aβ and tau pathology [265], implying that ApoE4 genotype might be a distinct risk factor for hyperexcitability. Young healthy humans who are ApoE4 carriers also show fMRI hyperactivity of the hippocampus [266]. Adeno-associated virus (AAV) vectors, and specifically the AAVrh.10-APOE2 vector, have shown promising results in mice and non-human primates in shifting the more detrimental ApoE4 genotype expression to ApoE2, with a single intracerebral injection resulting in decreased Aβ levels and amyloid plaque formation [267,268]. A pioneering phase 1 study with AAVrh.10-APOE2 vector is currently ongoing in ApoE4+ MCI and AD patients (Table 3). One possible implication therefore is that ApoE4+ individuals might be an important group to target for initial attempts to reduce brain hyperexcitability, but further data in humans are needed to confirm these promising preclinical data.

### 6.1. Diagnostic Tools

Whereas counteracting *hyper*excitability might be the optimal strategy in early phases of AD, preventing neuronal *hypo*excitability might be crucial in later phases [1]. Therefore, the timing of therapeutic strategies in different stages of AD (preclinical, prodromal, moderate, severe pathology) might need to be accounted for when designing clinical trials addressing neuronal hyperexcitability.

How would it be possible to stage a patient in vivo (Figure 1)? Hippocampal fMRI activation has gained attention as a marker of hyperexcitability, as it is increased in MCI patients compared to controls, and in early MCI compared to late MCIs, while AD patients typically show an hypoactivation pattern, thus suggesting this might reflect a temporal dynamic shift from hyper to hypoexcitability [23,269,270,271], (Figure 1). Notably, hippocampal fMRI hyperactivation has been found also in young, cognitively-intact presymptomatic individuals with the E280A PSEN1 mutation [272], in ApoE4+ individuals [273], and controls with a family history of AD [274], suggesting that it might be a possible signature of early preclinical neuronal dysfunction. It is also correlated with cortical thinning in brain regions typically associated with AD pathology [275], to longitudinal increased amyloid accumulation measured by PiB-PET and higher rates of cognitive decline [276], (Figure 1). “When” to treat seems, therefore, as soon as possible, given also that when hypoactivity is present, as shown by preclinical models, tau-related damage might already be irreversible [45].

Nevertheless, task-related fMRI hyperactivity is not a direct measure of epileptiform activity, so its interpretation as marker of epileptiform activity is still speculative. One key piece of evidence strengthening this link, however, is the finding that levetiracetam is able to counteract the hippocampal hyperactivation in MCI patients [22,185], implying that it is indeed reflecting underlying epileptiform activity.

What is the role of the most used tool to assess hyperexcitability in clinical practice, which is standard EEG? Areas of hyperexcitability might be limited to a small region such as the entorhinal cortex [155,170], and could coexist with hypoactive circuits, even in adjacent regions [45,269], making any changes difficult to detect by large scale surface EEG recordings [12]. Therefore, non-invasive scalp recording as provided by standard EEG might substantially underestimate brain hyperexcitability [277]. Moreover, epileptiform activity could be more prevalent during sleep [4,6] and therefore missed in routine clinical evaluations. Even if standard EEG abnormalities, as increased theta and delta activities, have shown a potential in tracking AD progression, longitudinal EEGs as are rarely used in clinical practice for AD staging [277], (Figure 1). A 24 h long-term monitoring by video-electroencephalography (LTM-EEG) telemetry has proven to increase the chances of uncovering subclinical epileptiform activity in AD patients [6]. Quantitative EEG (qEEG) analysis has also shown promise in detecting early neuronal dysfunction and to correlate with molecular and imaging biomarkers of the disease [278]. Another emerging technique to measure the disruption of neuronal fine tuning in AD is magnetoencephalography (MEG), which has several advantages over fMRI and EEG, combining high spatial and sub-millisecond temporal resolution [279]. MEG has been shown to outperform standard and prolonged EEG in detecting subclinical epileptiform activity in AD patients and controls [6]. It is able not only to detect localized patterns of reduced connectivity in AD patients [280], but also to predict future conversion from MCI to AD [281]. MEG can detect deficits of functional connectivity even in patients with subjective cognitive impairment, possibly providing a very early maker of the disease [282]. Intriguingly, metrics such as Synchronization Likelihood (SL), a measure of functional connectivity, could be increased in MCI patients and reduced in AD, possibly mirroring fMRI dynamics of initial hyper and subsequent hypoactivation [283].

Whether, however, these changes reflect an underlying hyperexcitable state, remains to be ascertained. Multiple MEG metrics show different trajectories alongside disease progression and MEG availability is still limited to a relatively smaller number of research centres [284]. One study supported the detection of Aβ-induced hyperexcitability in MCI patients, showing that Aβ-positive MCIs had increased alpha band power in medial frontal areas and increased delta band power, which correlated with disease progression within the AD continuum [285]. Even if some data suggest that MEG is able to record signal coming from the hippocampus, the decrease in MEG signal-to-noise ratio as a function of source depth implies that, as for surface EEG, its detection of subtle abnormalities in deep brain structures might be suboptimal [286]. A phase 2 clinical trial is ongoing to test the effect of levetiracetam on MEG signal changes in patients with MCI and AD (Table 1, levetiracetam (3)). Another clinical trial (NCT04131491) is currently recruiting to quantify subclinical epileptiform discharges and hippocampal hyperactivity with MEG, prolonged EEG and their impact on CSF biomarkers of AD.

### 6.2. Therapeutic Tools

Different non-pharmacological brain stimulation techniques such as transcranial magnetic stimulation (TMS), transcranial direct current stimulation (tDCS), transcranial alternating current stimulation (tACS), either alone or combined with EEG have been used either to diagnose or to treat brain hyperexcitability in AD through detection and modulation of LTP and LTD changes. Given the modulatory properties of TMS, and the possibility of detecting its impact at a granular temporal scale with EEG, these techniques have also been proposed as therapeutic tools to tune the brain’s excitatory state [287]. TMS protocols have been extensively used in AD for diagnostic and therapeutic purposes [288,289], particularly Theta burst Stimulation (TBS), which resembles the methods used for investigation of hippocampal plasticity [290], and its metrics of LTP reduction correlate with hippocampal-type cognitive impairment in AD [291]. Short latency afferent inhibition (SAI), which is a measure of cholinergic pathways’ integrity, shows that AD patients have impaired LTP-like cortical plasticity, with preservation of LTD [292]. TMS and TMS-EEG have been able to detect hyperexcitability in early stages of AD [293,294]. TMS-EEG with stimulation of the precuneus has been reported to ameliorate memory deficits and enhance beta oscillations in prodromal AD [295], and several trials in MCI or AD are currently ongoing (Table 4).

Several small studies with tDCS have shown some efficacy in enhancing memory function in AD patients, even if with conflicting results [296,297,298,299], (Table 4). tACS, with its ability to entrain or synchronize brain network oscillations, especially in the 40 Hz gamma frequency, is being explored as a therapeutic tool in AD disease [300], (Table 4). GammaSense stimulation, which delivers a LED light flashing at 40 Hz and auditory stimuli, has shown promise in different mouse models, including 5XFAD, APP/PS1, and wild type mice, with reduction of Aβ and tau levels and positive effect on microglia [301,302]. Positive effects in reducing amyloid load in auditory cortex and hippocampus, as well as a more widespread reduction of Aβ load, and improved spatial and recognition memory of 5XFAD mice, have been reported [303]. Moreover, reduced tau phosphorylation has been found in the P301S tauopathy model after GammaSense treatment [303]. Human studies applying GammaSense stimulation in MCI or AD are currently ongoing, though a small pilot study in 10 patients on 40 Hz light therapy had no effects on Aβ load [304], (Table 4). Some groups have also coupled TMS or tDCS with cognitive stimulation [305,306], (Table 4). Other devices, such as NeuroEM, based on Transcranial Electromagnetic Treatment (TEMT), seem to show promising results [307], and clinical trials to assess its efficacy are currently ongoing (Table 4). Alternative approaches are also being studied, such as temporal interference stimulation (TI), which can selectively modulate neurons in the deep brain structures in animal models and human prototypes [308,309], (Table 4). Intranasal delivery of near infrared (NIR) light via light emitting diodes, or photobiomodulation is also being tested in AD for its possible beneficial impact of mitochondrial function, and improvements in cognition, increased cerebral perfusion, and enhanced connectivity between the posterior cingulate cortex and lateral parietal nodes of the default-mode network after 12 weeks of treatment have been reported in a small pilot study [310], (Table 4).

All of these non-pharmacological brain stimulation techniques have their limitations. Some of these stimulation protocols have “history of seizure” as exclusion criterion, as they can lower seizure threshold [309], which might be extremely important in the context of increased hyperexcitability in AD patients. Moreover, the reported positive effects on cognition usually last only for few weeks after stimulation, and there is still little evidence for long-term cognitive benefit [311]. Besides these new approaches, which are available in the context of research, different pharmacological compounds such as ASMs have been used to address the question of “How” to treat brain hyperexcitability, targeting different steps of the excitotoxic cascade (Figure 2), and they remain at the moment the most reliable option.

## 7. Conclusions

Hyperexcitability, especially localized to the hippocampus, seems to be an early signature of neuronal and cognitive dysfunction in patients who are at risk of developing AD [269,270,271]. Preclinical models and human studies suggest that these changes reflect an early aberrant E > I (excitatory > inhibitory) imbalance, which is associated with Aβ synaptopathy, and fosters further reactive release of toxic compounds such as Aβ amyloid and tau [38,46,47]. These alterations might decrease during disease progression, as shown by the progressive tau induced neuronal silencing, i.e., E < I, and subsequent neurodegeneration in the later phases of the disease [1,38,45]. Therefore, there might be a very narrow window of opportunity to target brain hyperexcitability, which might need to be taken into account when designing clinical trials tackling hyperexcitability in AD.

Several ASMs have been proposed as a means of counteracting brain hyperexcitability in preclinical models of AD, as well as in patients [153], with levetiracetam showing promising results [183]. GABAergic modulation is also being explored, through repurposing of licensed medications; new GABA_A_ agonists and GABA_B_ antagonists; and innovative techniques such as gene and stem cell therapies [217].

Targeting cardiovascular risk factors, such as hypertension and diabetes, has been proposed to counteract the development of additional vascular lesions in AD patients, but also to help reduce brain hyperexcitability [235]. Clinical trials to tackle neuroinflammation, rather than systemic inflammation, through more tailored approaches are ongoing, as is work on gene editing via viral vectors to reduce the detrimental and pro-excitatory effects of ApoE4 genotype [267].

Non-pharmacological stimulation techniques have also been shown to enhance cognition in AD patients, at least in the short-term, by modulating brain hyperexcitability, and are being trialed for their possible long-term effects on AD pathological cascades.

One of the critical questions is what defines the best in vivo marker for hyperexcitability, as this would help stratify people with AD for clinical trials. In humans, fMRI has shown promising results in detecting early hippocampal alterations [269,271], but other approaches such as MEG or TMS-EEG might also be considered to measure brain hyperexcitability owing to their good temporal resolution and modulation potential [293,294].

Clinical trials targeting different molecular pathways that contribute to the genesis of such aberrant cortical function, as well as being of therapeutic relevance, offer insights on AD progression and how to potentially prevent the development of dementia in susceptible populations. Nevertheless, several clinical trials have failed so far in halting AD progression through modulation of possible targets of brain hyperexcitability, and multiple diagnostic and therapeutic challenges have yet to be overcome. Licensed drugs, as well as new strategies are being tested in cognitively healthy people at risk of developing AD, as well as in MCI and AD patients, mostly in early-prodromal phases. These upcoming trials could cast a light on the potential of brain fine-tuning, and possible disease modifying effects in AD.

## Figures and Tables

**Table 1:** Therapeutic strategies targeting Aβ and tau dependent pathways.

Class and Name	Mechanism	Population	Phase, NCT Number and Outcomes, Reference
**Aβ amyloid agents**			
Aducanumab	Passive immunotherapy	MCI ^1^ and Mild AD ^2^	Phase 3 (NCT02484547)-ongoing Phase 3 (NCT02477800)-ongoing
Bapineuzumab	Passive immunotherapy	(1) Mild-moderate AD ApoE4+ (2) Mild-moderate AD ApoE4−	(1) Phase 3 (NCT00667810)–failed [142] (2) Phase 3 (NCT00676143)–failed [142]
BAN2401	Passive immunotherapy	MCI and Mild AD	Phase 2 (NCT01767311)-ongoing Phase 3 (NCT03887455)-ongoing
Crenezumab	Passive immunotherapy	Mild AD	Phase 3 (NCT02670083)-terminated for lack of efficacy
Donanemab	Passive immunotherapy	Mild-moderate AD	Phase 2 (NCT03367403)-ongoing
Gantenerumab	Passive immunotherapy	Mild AD	Phase 3 (NCT03444870)-ongoing Phase 3 (NCT03443973)-ongoing
Ponezumab	Passive immunotherapy	Mild-moderate AD	Phase 2 (NCT00722046)–failed [143]
Solanezumab	Passive immunotherapy	MCI and mild AD	Phase 3 (NCT02760602)–terminated for lack of efficacy
**BACE-1 ^3^** **inhibitors**			
Atabecestat	BACE-1 inhibition	(1) Amyloid+ or ApoE4+ healthy subjects (2) Mild AD	(1) Phase 2/3 (NCT02569398)–failed [144] (2) Phase 2 (NCT02406027)–failed [145]
Elenbecestat	BACE-1 inhibition	Mild AD	Phase 3 (NCT03036280)-ongoing
Lanabecestat	BACE-1 inhibition	Early AD	Phase 2/3 (NCT02245737)-ongoing
Umibecestat	BACE-1 inhibition	ApoE4 + healthy subjects	Phase 2/3 (NCT03131453)–terminated for adverse cognitive effects
Verubecestat	BACE-1 inhibition	Mild AD	Phase 3 (NCT01953601)–failed [78]
**Tau agents**			
AADvac1	Active immunotherapy	(1) Mild-moderate AD (2) PPA ^4^	(1) Phase 2 (NCT0257925)–no effects on cognition, reduction NFL ^5^ and MRI ^6^ atrophy [101] (2) Phase 1 (NCT03174886)-ongoing
ACI-35	Active immunotherapy	MCI and Mild AD	Phase 2A (NCT04445831)-ongoing
Gosuranemab	Passive immunotherapy	MCI and Mild AD	Phase 2 (NCT03352557)-ongoing
Tilavonemab	Passive immunotherapy	MCI and Mild AD	Phase 2 (NCT02880956)-ongoing
Semorinemab	Passive immunotherapy	Mild-moderate AD	Phase 2 (NCT03289143)-ongoing
Zagotenemab	Passive immunotherapy	Mild-moderate AD	Phase 2 (NCT03518073)-ongoing Phase 2 (NCT03828747)-ongoing
Salsalate	Acetylation inhibitor	Mild-moderate AD	Phase 1 (NCT03277573)-ongoing
LMTM	Aggregator inhibitor	MCI and Mild AD	Phase 3 (NCT03446001)-ongoing
LY3372689	OGA inhibitors ^7^	Healthy participants	Phase 1 (NCT04392271)-ongoing
Davunetide	Microtubule stabilizers	MCI	Phase 2 (NCT00422981)–failed [105]
Rolipram	PDE4 ^8^ inhibitor	APP/PS1 mice	Preclinical Phase [107]
ASOs ^9^	MAPT ^10^ mRNA blockage	Mild AD	Phase 1/2 (NCT03186989)-ongoing
**GSK-3β ^11^ inhibitors**			
Tideglusib	GSK-3β inhibition	Mild-moderate AD	Phase 2a (NCT00948259)-trends for cognitive benefits [127] Phase 2b (NCT01350362)–failed [128]
Memantine	NMDA ^12^ NR2B antagonist	Moderate-severe AD	Licensed in moderate-severe AD
Ifenprodil	NMDA NR2B antagonist	Pentylenetetrazol (PTZ)-kindled rats	Preclinical Phase [129]
**Kinase inhibitors**			
Saracatinib	Src kinase inhibitor	Mild-moderate AD	Phase 2a (NCT02167256)–failed [139]
Masitinib	Tyrosine kinase inhibitor	Mild-moderate AD	Phase 2 (NCT00976118)-improvement in cognitive scores [140] Phase 3 (NCT01872598)-ongoing

^1^ MCI = mild cognitive impairment, ^2^ AD = Alzheimer’s disease, ^3^ BACE-1 = β-site amyloid precursor protein cleaving enzyme 1, ^4^ PPA = primary progressive aphasia, ^5^ NFL = neurofilament light chain, ^6^ MRI = magnetic resonance imaging, ^7^ OGA = O- GlcNAcase, ^8^ PDE4 = Phosphodiesterase E4, ^9^ ASOs = antisense oligonucleotides, ^10^ MAPT = microtubule associated protein tau, ^11^ GSK-3β = glycogen synthase kinase 3 beta, ^12^ NMDA = N-methyl-D-aspartate. Only the most advanced and recent trials are shown. Outcomes are based on information available in ClinicalTrials.gov.

**Table 2:** ASM and pro-epileptogenic compounds counteracting hyperexcitability.

Class and Name	Mechanism	Population	Phase, NCT Number and Outcomes, Reference
**ASMs** ^1^			
Levetiracetam	SV2A ^2^ binding	(1, 2, 3) Mild-moderate AD ^3^ (4, 5) Mild AD (6) MCI ^4^ (7) ApoE4+ healthy subjects	(1) Phase 2 (NCT04004702)-ongoing (2) Phase 2 (NCT03489044)-ongoing (3) Phase 2 (NCT02002819)-ongoing (4) Phase 2 (NCT03875638)-ongoing (5) Phase n/a (NCT01554683)-not reported (6) Phase 2 (NCT01044758)-not reported (7) Phase 2 (NCT03461861)-ongoing
Brivaracetam	SV2A binding	APP/PS1 mice	Preclinical Phase [182]
Lamotrigine	Na+ channel blocker	APP/PS1 mice	Preclinical Phase [169]
**NMDA modulators**			
Lithium	Downregulation of NMDA receptors, increasing GABAergic transmission, GSK-3β inhibition	MCI	Phase 4 (NCT03185208)-ongoing
BI425809	GlyT-1 and GlyT-2 ^5^ blockage	Mild AD	Phase 2 (NCT02788513)–failed [196]
AVP-786	NMDA antagonist	AD	Phase 3 (NCT04464564)-ongoing Phase 3 (NCT04408755)-ongoing
Troriluzole	GLT-1 ^6^ enhancement	AD	Phase 2/3 (NCT03605667)-ongoing
**GABAergic modulators**			
Zolpidem	GABA_A_ receptor agonist	AD	Phase 3 (NCT03075241)-ongoing
Tramiprosate	GABA_A_ receptor agonist and GABA_B_ receptor antagonist	MCI and AD	Nutritional supplement [203]
ALZ-801	GABA_A_ receptor agonist and GABA_B_ receptor antagonist	Healthy subjects	Phase 1 (NCT04585347)-not reported Phase 1 (NCT04157712)-not reported
SGS742	GABA_B_ receptor antagonist	(1) MCI (2) Mild-Moderate AD	(1) Phase 2 (NCT n/a)-improvements in memory [206] (2) Phase 2 (NCT00093951)–not reported
sAPP ^7^	GABA_B_R1α modulator	Thy1-GCaMP6s mice	Preclinical Phase [207]
KD ^8^	Ketone bodies production	(1, 3) AD (2) MCI and AD	(1) Phase n/a ^9^ (NCT03860792)-ongoing (2) Phase n/a (NCT03472664)-ongoing (3) Phase n/a (NCT02912936)-ongoing
Stem cells	Increase of GABAergic tone by restoring physiological cell phenotypes	(1, 4, 5) Mild-moderate AD (2) MCI (3) Mild AD (6) AD	(1) Phase 2 (NCT02833792)–ongoing (2) Phase 2 (NCT04228666)–ongoing (3) Phase 2 (NCT04482413)–ongoing (4) Phase 1/2 (NCT04388982)–ongoing (5) Phase 1/2 (NCT02899091)–ongoing (6) Phase 1/2 (NCT02054208)-not reported
Semagacestat	Nav1.1 channel enhancement	AD	Phase 3 (NCT00762411)–terminated for increased rates of skin cancer and lack of efficacy Phase 3 (NCT00594568)–failed [221] Phase 3 (NCT01035138)–terminated for increased rates of skin cancer and lack of efficacy

^1^ ASMs = antiseizure medications, ^2^ SV2A = synaptic vesicle glycoprotein, ^3^ AD = Alzheimer’s disease, ^4^ MCI = mild cognitive impairment, ^5^ GlyT-1 and 2 = glycine transporters, ^6^ GLT-1 = glutamate transporter-1, ^7^ sAPP = soluble amyloid precursor protein, ^8^ KD = ketogenic diet, ^9^ n/a = not applicable. Only the most advanced and recent trials are shown. Outcomes are based on information available in ClinicalTrials.gov.

**Table 3:** Therapeutics targeting modifiable risk factors for hyperexcitability.

Class and Name	Mechanism	Population	Phase, NCT Number and Outcomes, Reference
**Anti-hypertensive medication**			
Valsartan	Aβ amyloid reduction	Tg2576 mouse	Preclinical Phase [228]
**Anti-diabetic medication**			
Dapagliflozin	SGLT2 ^1^ inhibition	(1) Type 2 DM ^2^ (2) AD ^3^ with or without Type 2 DM	(1) Phase n/a ^4^ (NCT03961659)-ongoing (2) Phase 1 (NCT03801642)-ongoing
Intranasal insulin	Increase of brain glucose, reduce neuroinflammation and oxidative stress	(1) MCI and AD (2) MCI and AD (3) Healthy subjects or MCI (4) Healthy subjects or MCI	(1) Phase 2/3 (NCT01767909)–failed [238] (2) Phase 2 (NCT02503501)–terminated for lack of efficacy (3) Phase 2 (NCT03857321)–ongoing (4) Phase 3 (NCT04199767)–ongoing
**Anti-inflammatory drugs**			
Neflamapimod	p38-MAPK ^5^ kinase inhibitor	Mild AD	Phase 2 (NCT03435861)-ongoing
PTI-125	Filamin A inhibitor	Mild-moderate AD	Phase 2 (NCT04388254)-ongoing
**Gene therapy**			
AAVrh.10-APOE2	AAV ^6^ vectors	ApoE4+ MCI ^7^ or AD	Phase 1 (NCT03634007)-ongoing

^1^ SGLT2 = sodium-glucose cotransporter-2, ^2^ DM = diabetes mellitus, ^3^ AD = Alzheimer’s disease, ^4^ n/a = not applicable, ^5^ MAPK = mitogen-activated protein kinase, ^6^ AAV = adeno-associated virus, ^7^ MCI = mild cognitive impairment. Only the most advanced and recent trials are shown. Outcomes are based on information available in ClinicalTrials.gov.

**Table 4:** Non-pharmacological brain stimulation techniques for reducing brain hyperexcitability.

Class and Name	Mechanism	Population	Phase, NCT Number and Outcomes, Reference
TMS ^1^	Coil-induced depolarizing magnetic field	(1, 10) AD ^2^ (2) MCI ^3^ or AD (3, 4, 5, 6, 7, 8, 9, 12) Mild-moderate AD (11) PPA ^4^, MCI, AD	(1) Phase 2 (NCT00814697)–not reported (2) Phase 2 (NCT04555941)-ongoing (3) Phase n/a ^5^ (NCT03778151)-ongoing (4) Phase n/a (NCT04260724)-ongoing (5) Phase n/a (NCT03121066)-ongoing (6) Phase n/a (NCT02537496)-not reported (7) Phase n/a (NCT01481961)–ongoing (8) Phase n/a (NCT04263194)-ongoing (9) Phase n/a (NCT04294888)-ongoing (10) Phase n/a (NCT04562506)-not reported (11) Phase n/a (NCT04045990)-ongoing (12) Phase 4 (NCT02190084)-not reported
tDCS ^6^	Low direct electric currents	Mild-moderate AD	Phase n/a (NCT03288363)–ongoing Phase n/a (NCT04404153)–ongoing Phase n/a (NCT03313518)–cognitive improvement and increase in CSF ^7^ Aβ42 [312]
tACS ^8^	Sinusoidal, alternating low frequency currents	(1, 3) Mild-moderate AD (2) MCI (4) MCI and AD (5, 6) AD	(1) Phase n/a (NCT03290326)-not reported (2) Phase n/a (NCT04515433)-ongoing (3) Phase n/a (NCT03412604)-ongoing (4) Phase 1/2 (NCT03880240)-ongoing (5) Phase n/a (NCT03920826)-ongoing (6) Phase n/a (NCT04088643)-ongoing
GammaSense stimulation	40 Hz LED light and auditory stimuli	(1, 2) MCI and AD	(1) Phase n/a (NCT03556280)-ongoing (2) Phase n/a (NCT03661034)-ongoing
TMS/tDCS and cognitive stimulation	Brain stimulation and computer-based cognitive stimulation	(1, 2) Mild-moderate AD	(1) Phase n/a (NCT01825317)–not reported (2) Phase n/a (NCT01825330)–not reported
NeuroEM	Transcranial electromagnetic treatment (TEMT)	(1, 2) Mild-moderate AD	(1) Phase n/a (NCT03927040)-ongoing (2) Phase 1/2 (NCT04271163)-ongoing
Temporal interference stimulation (TI)	Two different electric fields via electrodes	Healthy subjects	Phase n/a (NCT03747601)-ongoing
Photobiomodulation	Intranasal delivery of near infrared light via diodes	(1) Healthy subjects at risk for AD (2) AD (3) AD	(1) Phase 2 (NCT04018092)-ongoing (2) Phase n/a (NCT03405662)-ongoing (3) Phase n/a (NCT03160027)–improvements in cognition, cerebral perfusion and brain connectivity [310]

^1^ TMS = Transcranial magnetic stimulation =, ^2^ AD = Alzheimer’s disease, ^3^ MCI = mild cognitive impairment, ^4^ PPA = primary progressive aphasia, ^5^ n/a = not applicable, ^6^ tDCS = transcranial direct current stimulation, ^7^ CSF = cerebrospinal fluid, ^8^ tACS = transcranial alternating current stimulation. Only the most advanced and recent trials are shown. Outcomes are based on information available in ClinicalTrials.gov.

## Abbreviations

AD	Alzheimer’s disease
ASM	Antiseizure medication
MRI	Magnetic resonance imaging
EEG	Electroencephalogram
E/I	Excitation and inhibition
APP	Amyloid precursor protein
PSEN1	Presenilin-1 (gene mutation)
PS1	Presenilin-1 (protein)
LTP	Long-term potentiation
LTD	Long-term depression
hiPSC	Human induced pluripotent stem cell
CSF	Cerebrospinal fluid
PiB	Pittsburgh B compound amyloid ligand
BACE-1	β-site amyloid precursor protein cleaving enzyme 1
NFT	Neurofibrillary tangles
NFL	Neurofilament light chain
OGA	O- GlcNAcase
MCI	Mild cognitive impairment
PDE4	Phosphodiesterase E4
BBB	Blood–brain barrier
ASOs	Antisense oligonucleotides
MTBR	Microtubule binding region
GSK-3β	Glycogen synthase kinase 3 beta
GLT-1	Glutamate transporter-1
VGLT-1	Vesicular glutamate transporter 1
SV2A	Synaptic vesicle glycoprotein 2A
PSEN2	Presenilin-2
fMRI	Functional magnetic resonance imaging
GlyT-1	Glycine transporter 1
GlyT-2	Glycine transporter 2
KD	Ketogenic diet
SVD	Small vessel cerebrovascular disease
SGLT-2	Sodium-glucose cotransporter-2
DM	Diabetes mellitus
AAV	Adeno-associated virus
TMS	Transcranial magnetic stimulation
tDCS	Transcranial direct current stimulation
tACS	Transcranial alternating current stimulation
